# A New Paired Associative Stimulation Protocol with High-Frequency Peripheral Component and High-Intensity 20 Hz Repetitive Transcranial Magnetic Stimulation—A Pilot Study

**DOI:** 10.3390/ijerph182111224

**Published:** 2021-10-26

**Authors:** Sabin Sathyan, Aleksandra Tolmacheva, Sergei Tugin, Jyrki P. Mäkelä, Anastasia Shulga, Pantelis Lioumis

**Affiliations:** 1 Research Group of Computational Electromechanics, Department of Electrical Engineering and Automation, Aalto University, 02150 Espoo, Finland; 2 BioMag Laboratory, HUS Medical Imaging Center, University of Helsinki and Helsinki University Hospital, 00280 Helsinki, Finland; a.tolmacheva12@gmail.com (A.T.); sergei.tugin@aalto.fi (S.T.); jyrki.makela@hus.fi (J.P.M.); anastasia.shulga@helsinki.fi (A.S.); 3 Department of Neuroscience and Biomedical Engineering, Aalto University School of Science, 02150 Helsinki, Finland; 4 Department of Physical and Rehabilitation Medicine, Helsinki University Hospital, 00280 Helsinki, Finland

**Keywords:** corticospinal tract, long interval cortical inhibition, neuroplasticity, paired associative stimulation, peripheral nerve stimulation, primary motor cortex, spinal cord injury, transcranial magnetic stimulation

## Abstract

Paired associative stimulation (PAS) is a stimulation technique combining transcranial magnetic stimulation (TMS) and peripheral nerve stimulation (PNS) that can induce plastic changes in the human motor system. A PAS protocol consisting of a high-intensity single TMS pulse given at 100% of stimulator output (SO) and high-frequency 100-Hz PNS train, or “the high-PAS” was designed to promote corticomotoneuronal synapses. Such PAS, applied as a long-term intervention, has demonstrated therapeutic efficacy in spinal cord injury (SCI) patients. Adding a second TMS pulse, however, rendered this protocol inhibitory. The current study sought for more effective PAS parameters. Here, we added a third TMS pulse, i.e., a 20-Hz rTMS (three pulses at 96% SO) combined with high-frequency PNS (six pulses at 100 Hz). We examined the ability of the proposed stimulation paradigm to induce the potentiation of motor-evoked potentials (MEPs) in five human subjects and described the safety and tolerability of the new protocol in these subjects. In this study, rTMS alone was used as a control. In addition, we compared the efficacy of the new protocol in five subjects with two PAS protocols consisting of PNS trains of six pulses at 100 Hz combined with (a) single 100% SO TMS pulses (high-PAS) and (b) a 20-Hz rTMS at a lower intensity (three pulses at 120% RMT). The MEPs were measured immediately after, and 30 and 60 min after the stimulation. Although at 0 and 30 min there was no significant difference in the induced MEP potentiation between the new PAS protocol and the rTMS control, the MEP potentiation remained significantly higher at 60 min after the new PAS than after rTMS alone. At 60 min, the new protocol was also more effective than the two other PAS protocols. The new protocol caused strong involuntary twitches in three subjects and, therefore, its further characterization is needed before introducing it for clinical research. Additionally, its mechanism plausibly differs from PAS with high-frequency PNS that has been used in SCI patients.

## 1. Introduction

Paired Associative Stimulation (PAS), first introduced by Stefan et al. [1], is a non-invasive neuromodulation paradigm. It encompasses the repeated pairing of electrical stimuli on a peripheral nerve, innervating the target muscle and transcranial magnetic stimulation (TMS) of the primary motor cortex (M1) that has direct corticospinal projections to the target muscle. The PAS induces spike timing dependent plasticity (STDP) in the corticospinal tract and strengthens the cortico-muscular connections [1]. In PAS, the descending volleys from M1 induced by TMS are timed to coincide with the antidromic volleys elicited by PNS. The continued pairing of these two stimuli strengthens the synaptic connections and produces long-term potentiation (LTP)-like plasticity in the corticospinal tract [2]. The PAS-induced changes of the motor evoked potential (MEP) amplitudes depend on the interval between the TMS and PNS pulses [3,4,5]. This inter-stimulus interval (ISI) determines the effect of PAS, which may produce either MEP potentiation or depression. PAS can also induce STDP in the spinal cord [6,7,8,9,10,11]. Several studies report the effect of PAS in different muscles in the human upper limb [12,13,14,15,16,17,18,19] and lower limb muscles [10,20,21,22,23,24]. In [16], a repetitive PAS paradigm is presented that produced a long-lasting increase in corticospinal excitability by sensorimotor disinhibition. PAS needs an individualized ISI to elicit substantial effects in the lower limb muscles [22,23].

PAS can be employed in the rehabilitation of incomplete chronic spinal cord injury (SCI) patients. It enhances the synaptic strength at the spinal cord level and can improve motor control over the paralyzed muscles [8,25]. Spinal PAS improves the corticospinal-motoneuronal synapse excitability and increases the muscle voluntary force in healthy individuals and in patients with partial SCI [6,8,10,25,26,27,28]. A series of PAS with single TMS pulses and a 100-Hz PNS train delivered at 0.2 Hz augments the strength of the paretic or paralytic muscles in incomplete SCI patients for at least several months [29,30,31].

The efficacy of a PAS protocol depends, e.g., on the number and frequency of pulses in TMS and PNS, and the ISI between the two stimuli. In addition, individual characteristics, pre-PAS activities and the time of the day when PAS is delivered influence the PAS outcome [32]. Mezes et al. tested a 0.2-Hz PAS with different TMS and PNS frequencies in eliciting MEP potentiation [33]. Two pulses of rTMS at 20 Hz delivered at 96% of the maximum stimulator output (MSO), combined with a 100-Hz PNS, somewhat surprisingly, suppressed the MEPs. The motor system of SCI patients undergoes plastic reorganization after the injury. The cortical motor representations and excitability of paralyzed muscles are reduced in SCI patients [34]. Inclusion of facilitatory rTMS in our PAS protocol may strengthen the PAS effect by enhancing the cortical and spinal excitability. The “high-PAS” with single 100% SO TMS pulses is effective in a wide range of patients with incomplete SCI. Several other variations (0.4-Hz PAS vs. 0.2-Hz PAS, 200-Hz and 400-HZ PNS vs. 100-Hz PNS, two TMS pulses vs. single pulse TMS) are less efficient than the high-PAS with single pulse TMS and 100-Hz PNS [33]. To further optimize PAS to achieve maximum efficacy in patients with SCI, this study explores the potential of an additional increase in the number of TMS pulses in PAS. No serious adverse effects were observed in our previous studies on high-PAS and its variations, and the devices for high-PAS have a well-characterized safety profile. In this work, we used three rTMS pulses at a 20-Hz frequency, two of those timed to coincide with two pulses of the PNS train in PAS. The new PAS protocol was tested with rTMS at 96% SO and at an SO equal to 120% of the resting motor threshold (RMT) of individual subjects. In addition, we compared the efficacy of the new protocols with our high-PAS (single TMS at 100% and PNS train). As a control, we also delivered the same three-pulse 96% SO rTMS alone in the same subjects. The PNS alone was not used as a control, because a 100-Hz PNS alone does not induce MEP potentiation [35,36,37]. Besides the efficacy of the new PAS, we intended to examine the safety and feasibility of the protocols.

To better understand the observed PAS effects, we also studied the effect of PAS on the long interval cortical inhibition (LICI) elicited by paired pulse TMS (ppTMS). To elicit the LICI, a conditioning stimulus (CS) and a test stimulus (TS) at the same intensity are delivered to the same cortical region with an ISI ranging between 50 and 200 ms [38,39,40]. The ppTMS can either facilitate or inhibit MEPs, depending on the intensities of CS and TS and the ISI between them [41]. The effect of the three PAS protocols on the LICI aids in determining whether PAS exerts its effect primarily on the cortical or spinal level.

## 2. Materials and Methods

### 2.1. Participants

The study was approved by the Ethics Committee of Helsinki University Hospital (HUS/1280/2016). Five healthy participants without any contraindications for TMS were recruited and signed an informed consent form before participating in the experiments. All subjects were right-handed; three were male and two were female; the age range was 27–50 years and the mean age 35 years. 

### 2.2. Transcranial Magnetic Stimulation

Nexstim eXimia TMS stimulator (Nexstim Ltd., Helsinki., Finland) with an integrated EMG device was used for generating TMS pulses using a figure-of-eight coil. In the experiments, we employed MRI-guided TMS navigation (nTMS; Navigated brain stimulation 4.3 (NBS 4.3), Nexstim Ltd., Helsinki, Finland) using 3D MRI models of individual 3T T1/T2 images. This enables accurate navigation of the coil and localization of M1, and the TMS-induced electric field can be targeted to the same cortical location with high reproducibility. The TMS was targeted to activate right abductor hallucis (AH). To activate the AH “hotspot” in M1, the TMS coil was placed over the left M1. The motor cortex mapping was conducted by delivering TMS pulses to the whole motor representation area of the distal lower limb. The AH hotspot was determined by locating the spot and coil orientation that produced largest MEPs from the right AH activated with plantar flexion. TMS pulses were given over the hotspot, and the minimum TMS intensity that evoked an MEP of >50 µV in 5 out of 10 trials was selected as the RMT. The average of 10 MEPs elicited at an interval of 3.3 s at 120% RMT was used for calculating the ISI between the TMS and PNS pulses during PAS. 

### 2.3. Peripheral Nerve Stimulation

PNS and F-response studies were performed with a Dantec Keypoint® electroneuromyography device (Natus Medical Inc., Pleasanton, CA, USA) and surface electrodes (Neuroline 720, AMBU A/S, Ballerup, Denmark). For tibial nerve stimulation, two electrodes were positioned between the medial malleolus and the Achilles tendon. To alleviate the sensations produced by the electric pulses, 2.5% lidocaine/prilocaine ointment (EMLA®) cream was applied locally at the stimulation site. For F-response measurements, two recording electrodes were placed over the belly of the AH muscle and the reference electrode was located on the medial side of the hallux. From ten F-responses recorded with a single 0.2-ms stimulation at a supramaximal intensity, the response with the shortest F-latency was used in the ISI calculation. The individual minimum intensity evoking F-responses was identified by applying square wave pulses of 1 ms and this intensity was used in the nerve stimulation during the PAS. 

### 2.4. Paired Associative Stimulation Protocols

**Figure 1:**
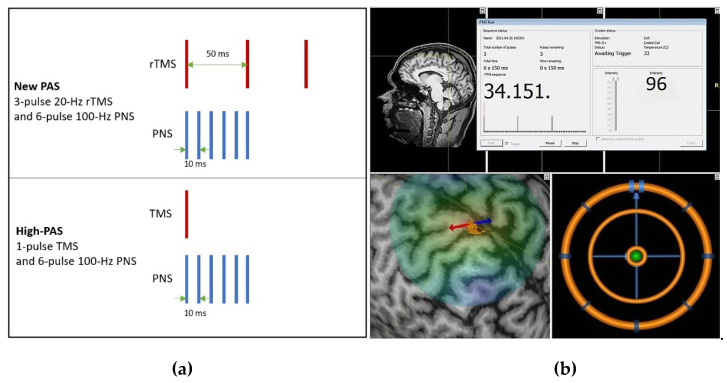
(**a**) Schematic representation of the new 3-pulse PAS and the high-PAS; (**b**) The nTMS interface showing the TMS pulse focused on the hotspot over M1.

During PAS, the TMS and PNS were triggered by Presentation ® software (Neurobehavioral Systems Inc., Albany, NY, USA). In the stimulation, each TMS pulse or train of 3 pulses was paired with the PNS train, and the ISI between the TMS and PNS was calculated using a formula (F - MEPavg) [10,36]. The MSO available from the Nexstim stimulator for a 20-Hz rTMS was 96%. The three TMS pulses were given at 96% MSO due to inbuilt safety limitations of the TMS device, based on the calculations in [42]. In this study, we tested three different PAS protocols. The new 3-pulse PAS consisted of three rTMS pulses delivered at 20 Hz with an intensity of 96% of the SO, combined with a 100-Hz PNS. In addition, we compared the efficacy of this 3-pulse PAS with a PAS protocol of a 100-Hz PNS combined with (a) a single 100% SO TMS (high-PAS, earlier used in SCI patient studies), and (b) a lower intensity 20-Hz 3-pulse rTMS at 120% of the RMT. We also delivered the same 20-Hz 3-pulse 96% SO rTMS alone as a control in the same subjects. In a single PAS experiment, a total of 240 rTMS bursts were delivered once every 5 s. Figure 1 shows the schematic representation of our high-PAS and the new 3-pulse PAS. The nTMS system user interface at an arbitrary stimulation point, where the coil orientation is focused over the hotspot, is also shown.

Corticospinal excitability was measured with MEPs. In all experiments, 30 MEPs elicited by TMS delivered to the AH hotspot at 120% RMT with an interval of 3.3 s were averaged for the amplitude and latency measurements. MEPs were measured before the PAS stimulation (pre-PAS MEP), immediately (0 min), 30 min and 60 min after the PAS. The percentage ratio was calculated by dividing the post-simulation MEP (in µV) with the pre-stimulation MEP. The baseline MEP value was 100%. Values above 100% indicated MEP facilitation, and values below 100 % indicated MEP suppression. The stimulation parameters used for the subjects are given in Table 1.

### 2.5. Long Intracortical Inhibition Protocol

The LICI protocol was applied with two TMS pulses delivered with a 100-ms ISI once every 3.3 s. The LICI was estimated immediately after the MEP measurements that were used to evaluate the PAS effects. The US and CS intensities were both 120% of the RMT. To evaluate the LICI effect, the MEP amplitude induced by CS was divided by the amplitude of US and multiplied by 100. MEPs recorded with the LICI protocol were analyzed with a custom-made script in Matlab 2021a (MathWorks, Inc., Natick, MA, USA). 

### 2.6. Statistical Analysis

Statistical analysis of the experimental data was carried out using IBM SPSS software version 28.0. Wilcoxon signed rank test was used for analyzing the significance of the results of different protocols between the subjects and Friedman test was used for comparing the average outcomes of different protocols. Wilcoxon signed rank test examined the statistical significance of MEP potentiations produced by each protocol in the sample (*n* = 5). The test was conducted by comparing the post-stimulation MEP values with pre-stimulation MEP values as related samples, in each protocol. The Friedman test was performed to analyze the statistical significance of the post-stimulation long-term potentiation of tested protocol “PAS rTMS 3-pulse 96% SO” compared with the three other protocols. To study the effect of the proposed protocol relative to the three other protocols, the MEP potentiations of five subjects elicited by four protocols at three post-stimulation measurement points were compared. This test analyzed the significance of the difference between the effects of different protocols (*n* = 4).

## 3. Results

### 3.1. Motor Evoked Potentials

**Figure 2:**
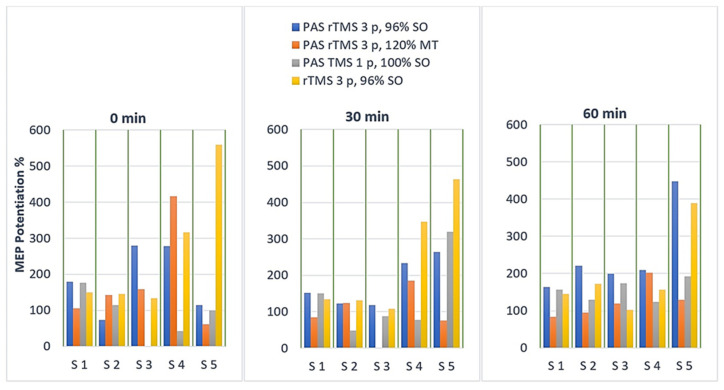
MEP potentiations elicited by the four different protocols at three post-stimulation time-points (immediately after the stimulation at 0 min, after 30 min and after 60 min). The MEP measurements of subject 3 were omitted in two sessions due to a technical error.

Figure 2 shows the individual MEP values of the five subjects at three measurement points. The new three-pulse protocol (PAS rTMS three-pulse 96% SO) 60 min after the stimulation surpasses the results of every other protocol in all the subjects. PAS with a three-pulse rTMS at 120% RMT potentiated MEPs at 60 min weakly in two subjects, although it increased the MEP amplitudes immediately after the stimulation in three subjects. PAS with the one-pulse TMS potentiated MEPs in all subjects at 60 min, but less than the new three-pulse PAS protocol. The three-pulse rTMS alone also potentiated MEPs at 60 min in all the subjects. Moreover, it produced stronger MEPs than the new three-pulse PAS protocol in three of the five subjects immediately after the stimulation and at 30 min. The range of long-term MEP potentiation after 60 min produced by the new two-pulse PAS was 163–447%, and the median value was 209%.

**Figure 3:**
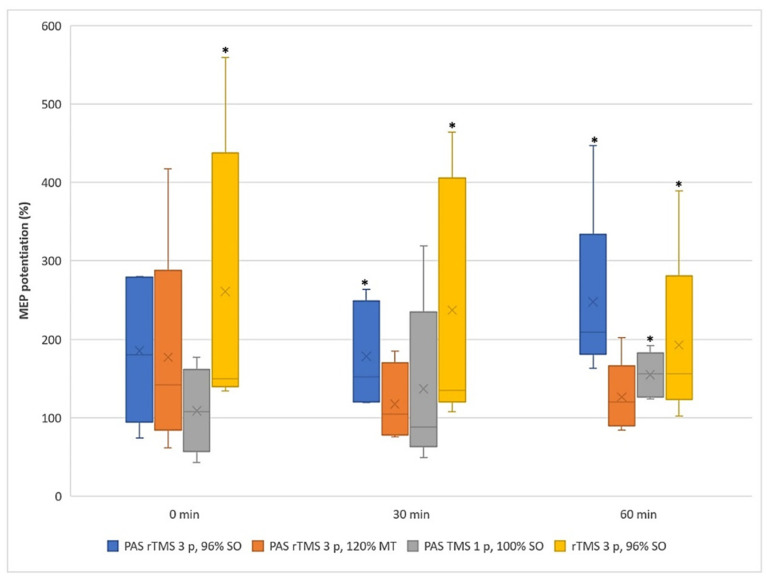
The median values of MEP potentiations among the subjects (*n* = 5) produced by four tested protocols at three post-stimulation measurement points. The bars with * sign represent the statistically significant outcomes (see Table 2). Comparing the median is more relevant in this study because the data are skewed and not normally distributed, as depicted in Figure 2.

Figure 3 illustrates the median of MEP potentiations. The new three-pulse PAS protocol potentiated MEPs significantly when compared to the pre-PAS values at 30 min (median/SE = 178 ± 30%, *p* = 0.043) and at 60 min (median/SE = 209 ± 52%, *p* = 0.043). The three-pulse rTMS only protocol potentiated MEPs significantly compared to pre-PAS at all three time points. The PAS protocol with a single TMS pulse induced significant MEP potentiation compared to the pre-PAS level at 60 min (see Table 2).

The Friedman test resulted in a *p*-value of 0.021 at 60 min post-stimulation. The new three-pulse PAS had a mean rank of 4.0, whereas the mean rank of the other protocols was 1.6. Thus, the results shown in Figure 3 are statistically significant at 60 min. The mean rank of the proposed new three-pulse PAS protocol clearly exceeds the mean ranks of the other protocols, suggesting that the new three-pulse PAS protocol is most likely to potentiate MEPs.

### 3.2. Long Interval Cortical Inhibition

**Figure 4:**
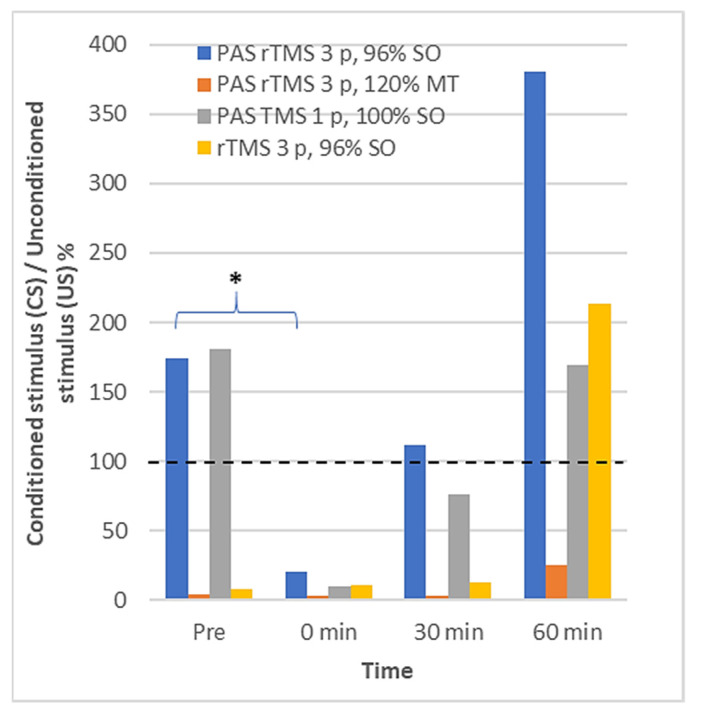
The median values of conditioned stimulus (CS) amplitude divided by unconditioned stimulus (US) amplitude among all subjects (*n* = 5) produced by four tested protocols at the pre-stimulation and three post-stimulation measurement points. The values below 100% indicate LICI. Values higher than 100% indicate that the MEP amplitude induced by CS was facilitated. The bars with * sign represent the statistically significant outcomes (see Table 3).

Figure 4 illustrates the grand average of the EMG responses to the LICI protocol across the five subjects. Only the new PAS rTMS with three pulses and 96% SO significantly modulated the LICI at 0 min in comparison to the pre-PAS LICI (Table 3). At 0 min, a clear LICI was observed after all four protocols. During the follow-up, the LICI was observed systematically only after the protocol with a three-pulse TMS at 120% RMT. After the other three protocols, the LICI wore out during the follow-up, and the MEPs to conditioned pulses at 60 min were enhanced, not suppressed as expected in the LICI. The effect was clearest for the protocols with the three-pulse TMS.

### 3.3. Safety and Tolerability of the New Protocol 

The train of three rTMS pulses within PAS produced high involuntary twitches/reflex reactions in most participants. However, no pain or additional side effects were observed during or after the stimulation. The reflex reactions caused twitching and jolting movements in three of the five subjects during the TMS pulse delivery during the three-pulse PAS. For the subjects with a higher RMT, the reflex reactions were smaller, and it caused only minor twitching of the foot. The rTMS alone protocol caused high involuntary twitches in only one of the five subjects. The navigation of the TMS coil and keeping the coil in the correct position throughout the stimulation was difficult with subjects having strong twitches.

## 4. Discussion

### 4.1. Efficacy and Safety of the Proposed PAS Paradigm

The proposed new three-pulse PAS paradigm induced the strongest MEP potentiation 60 min after the stimulation. Previous trials on variants of PAS with a high-frequency peripheral component [33] combined with two rTMS pulses suppressed MEPs. The second TMS pulse 50 ms after the first one induced a cortical inhibition, probably because of the cortical LICI [43]. In our study, the addition of a third TMS pulse potentiated MEPs, suggesting that the third pulse eliminates the LICI effect, and facilitates MEPs even more.

A single pulse TMS given at 100% SO or a 100-Hz PNS alone do not potentiate MEPs and, hence, the potentiation observed by combining them is the result of this dual stimulation [36]. Our three-pulse rTMS without PNS also facilitated MEPs significantly. However, adding PNS to the three-pulse TMS protocol prolonged and enhanced the MEP potentiation. The MEP facilitation after introducing a third pulse can be attributed to mechanisms similar to the triple stimulation technique (TST) [44,45]. MEP size is influenced by the number of stimulated motor neurons in the spinal cord, the number of neurons discharging more than one time to TMS and the synchronization of the discharges due to TMS [46]. In the TST, using three TMS pulses and two stimulations on different nerves [47] or one for the foot [48], the MEP amplification is caused by the desynchronization of motor neuron discharges to TMS. A similar effect may have been caused by our augmented PAS with three TMS pulses, leading to the elimination of the influence of motor neuron discharges on MEPs. 

In high-PAS, the dual stimulation is essential for plasticity induction, since neither PNS nor the single-pulse TMS component alone induces MEP potentiation. The high-PAS is designed in a way that the timing of the stimuli is optimized to induce the effect at the level of the spinal cord; however, additional cortical effects are also possible [36]. In the new protocol, the first pulse of a 20-Hz rTMS train is coupled to the first pulse of the 100-Hz PNS train as in high-PAS, the second pulse is coupled to the last pulse of the train, and the third pulse does not match with concomitant peripheral pulses. Importantly, the rTMS component alone induced strong MEP potentiation, and PAS potentiation was stronger than rTMS alone only at 60 min after the stimulation. An otherwise similar protocol but without the third pulse led to a strong MEP depression [33]. Since this two-pulse inhibitory protocol overrode the excitatory action of the high-PAS protocol, and here the excitatory three-pulse protocol led to MEP potentiation, we suggest that the cortical effect of the TMS component is superposed on the effect of high-PAS. Therefore, in the current protocol, the main source of the additional MEP potentiation is due to the rTMS component, and the effect occurs mainly in the cortex. The present data do not yet provide the information on the exact mechanism of why the addition of PNS enhanced the effect of rTMS at 60 min. Non-navigated 20-Hz rTMS delivered in 2-s trains to the foot representation area of the SCI patients daily for 4 weeks somewhat enhances the maximum voluntary contraction strength of the lower limb muscles when combined with resistance training but does not result in improved gait function [49]. The effects of PNS in combination with 20-Hz rTMS in SCI rehabilitation is not known.

The three-pulse protocol with 120% RMT TMS also resulted in long-term MEP potentiation in three of the five subjects. This indicates that the TMS intensity does play a role in PAS rehabilitation protocols and supports the use of 100% SO in the PAS with high-frequency PNS, which has led to positive therapeutic outcomes in spinal cord injury patients [30,31,35,36,50]. 

The motor responses in individual subjects after PAS sessions can be influenced by age, alertness, focus of attention at the time of the experiments and genetic factors. This study presents the experimental results from only five subjects with an age range of 27–50 years. The variability of the RMT of the foot muscles is larger than that of the hand muscles due to variable depth of the foot representation in the interhemispheric fissure. In general, the differences in MEP potentiation among the participants are in line with the high variability in responsiveness to PAS [32].

The tolerability to the sensations elicited by rTMS pulse trains needs to be considered before the new three-pulse TMS protocol can be applied in clinical research. Increasing the number of healthy subjects and further comparing this protocol to rTMS-only to better understand its mechanism is a prerequisite for applying it to patients. Since this is a pilot study in five healthy subjects only, the data are too preliminary to speculate its clinical feasibility. Presumably, the strongest MEP changes induced by this protocol result from changes of motor cortex function, and these data cannot be directly extrapolated on the clinical results obtained with the high-PAS protocol in spinal cord injury patients [36]. Additionally, strong muscle twitches experienced by healthy subjects preclude, at this stage, preliminary experiments in spinal cord injury patients who usually have problems connected to the overuse of musculoskeletal structures with residual neural activity. Further research of this protocol in healthy subjects will be useful in identifying its site of action as well as the patient groups who could benefit from it.

### 4.2. Effects on LICI

The LICI was observed immediately after all the PAS protocols. At 60 min, the LICI was still evident after the protocol with a three-pulse TMS at 120% RMT. In protocols with high-intensity TMS, the facilitation of MEPs to conditioned stimuli was observed at 60 min instead of suppression. The results suggest that high-intensity TMS leads to the eventual facilitation of the cortical excitability in some subjects. 

The LICI is related to the neuromodulators that are influenced by the GABA_B_ transmission [41]. The observed results suggest that PAS with high-intensity TMS has an effect on the neurons related to the GABA_B_ neuronal circuits [51].

The LICI results need to be considered carefully. First, the data from two subjects demonstrated a very strong LICI for all the time intervals after all PAS protocols. Additionally, we could not observe the LICI in three subjects before the PAS protocols. At this time, the GABA_B_ circuits have not been stimulated and we expected to see the LICI as reported by Claus and Valls-Sole [40,52]. Therefore, the stimulation parameters might not have been optimal for inducing a leg LICI, and the results remain tentative. 

## 5. Conclusions

The main objective of this study was to investigate whether the PAS paradigm used in SCI rehabilitation consisting of a 100-Hz PNS and single TMS pulses given at 100% SO can be made more efficient. The new three-pulse TMS protocol is more effective in potentiating MEPs at 60 min after the stimulation. The same three-pulse rTMS alone protocol also leads to similar effects. Therefore, the mechanism of this new PAS variant is most probably different from the protocol used in SCI rehabilitation [36]. It is noteworthy that, while the combination of two TMS pulses and a 100-Hz PNS led to depression [33], here the combination of three pulses of a 20-Hz rTMS and a 100-Hz PNS has led to strong and durable potentiation. TMS-induced involuntary twitches were stronger when the new three-pulse TMS protocol was applied, and hence its feasibility and safety need to be further evaluated. The strength and stability of MEP potentiation induced by it deserves further investigation. The main focus of this study was to test the safety and feasibility of the proposed PAS protocol on healthy volunteers and is limited by a small number of subjects. We will conduct further studies with a larger sample size to understand the significance and relevance of the results.

## Figures and Tables

**Table 1:** Stimulation parameters used for individual subjects.

Subjects	RMT (% SO)	PNS Intensity (mA)	F Latency (ms)	MEP Latency (ms)	ISI (ms)
**S1**	60	7	48.3	44.8	4
**S2**	55	9	54.5	42.7	12
**S3**	77	16	55.0	48.0	7
**S4**	64	13	54.0	43.6	10
**S5**	75	7	58.0	42.3	16

**Table 2:** The *p*-values and standard error of 4 protocols at three measurement points from Wilcoxon test.

Measurement Time	PAS rTMS 3p 96% SO	PAS rTMS 3p1 20% MT	PAS TMS 1p 100% SO	rTMS 3p 96% SO
**Post 60 min**	*p* = 0.043, SE = ±52%	*p* = 0.500, SE = ±21%	*p* = 0.043, SE = ±13%	*p* = 0.043, SE = ±50%
**Post 30 min**	*p* = 0.043, SE = ±30%	*p* = 0.465, SE = ±25%	*p* = 0.893, SE = ±49%	*p* = 0.043, SE = ±71%
**Post 0 min**	*p* = 0.345, SE = ±42%	*p* = 0.138, SE = ±62%	*p* = 0.285, SE = ±28%	*p* = 0.043, SE = ±82%

**Table 3:** The *p*-values for LICI of different protocols at three measurement points from Wilcoxon signed rank test.

Measurement Time	PAS rTMS 3p 96% SO	PAS rTMS 3p 120% MT	PAS TMS 1p 100% SO	rTMS 3p 96% SO
**Post 60 min**	*p* = 0.345	*p* = 0.144	*p* = 0. 465	*p* = 0.5
**Post 30 min**	*p* = 0.225	*p* = 0.068	*p* = 0. 465	*p* = 0.5
**Post 0 min**	*p* = 0.043*	*p* = 0.465	*p* = 0. 465	*p* = 0.5

## Data Availability

Not applicable.
